# Oocyte ageing and epigenetics

**DOI:** 10.1530/REP-14-0242

**Published:** 2015-03

**Authors:** Zhao-Jia Ge, Heide Schatten, Cui-Lian Zhang, Qing-Yuan Sun

**Affiliations:** Reproductive Medicine Center, Henan Provincial People's Hospital, #7 Weiwu Road, Jinshui District, Zhengzhou, Henan Province, 450003, People's Republic of China; State Key Laboratory of Reproductive Biology, Institute of Zoology, Chinese Academy of Sciences, #1 Beichen West Road, Chaoyang District, Beijing, 100101, People's Republic of China; Reproductive Medicine Center, People's Hospital of Zhengzhou University, Zhengzhou, Henan Province, 450003, People's Republic of China; Department of Veterinary Pathobiology, University of Missouri, Columbia, Missouri, 65211, USA

## Abstract

It has become a current social trend for women to delay childbearing. However, the quality of oocytes from older females is compromised and the pregnancy rate of older women is lower. With the increased rate of delayed childbearing, it is becoming more and more crucial to understand the mechanisms underlying the compromised quality of oocytes from older women, including mitochondrial dysfunctions, aneuploidy and epigenetic changes. Establishing proper epigenetic modifications during oogenesis and early embryo development is an important aspect in reproduction. The reprogramming process may be influenced by external and internal factors that result in improper epigenetic changes in germ cells. Furthermore, germ cell epigenetic changes might be inherited by the next generations. In this review, we briefly summarise the effects of ageing on oocyte quality. We focus on discussing the relationship between ageing and epigenetic modifications, highlighting the epigenetic changes in oocytes from advanced-age females and in post-ovulatory aged oocytes as well as the possible underlying mechanisms.

## Introduction

**Figure 1:**
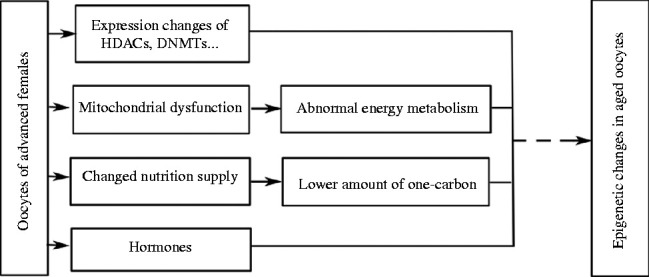
Schematics about relationship between epigenetic changes in oocytes and advanced maternal age. Advanced maternal age causes decrease in oocyte quality, including expression of HDACs and DNMTs, mitochondrial dysfunction, abnormal nutrition supply, changed levels of hormones and others. Thus, many pathways in oocytes may be disrupted, which may be involved in the process of establishing proper epigenetic modification in oocytes.

The average age of women bearing children has increased by ∼5 years in the past several decades (te Velde & Pearson 2002). However, advanced maternal age has deleterious effects on oocyte maturation and embryonic development, for which the decreasing oocyte quality with ageing may play a key role (Henderson & Edwards 1968, Tarin *et al*. 1998*a*, Sher *et al*. 2007, Simsek-Duran *et al*. 2013, Di Emidio *et al*. 2014, Rambags *et al*. 2014). These aspects have been well reviewed in our previous publications (Miao *et al*. 2009, Qiao *et al*. 2014); therefore, we summarise what is known on epigenetic alterations and relate changes in the epigenome with alterations in gene expression, mitochondrial dysfunction, nutrition, and hormonal homeostasis (Fig. 1).

The risk of childlessness, stillbirth and multiple births for daughters born by aged mothers (≥40 years) is increased when compared with daughters born to young mothers (24–30 years of age) (Smits *et al*. 2002, Sekhon *et al*. 2014, Selemani *et al*. 2014). Several studies have indicated that maternal age is negatively correlated with the health of the offspring (Rocca *et al*. 1991, Kemkes-Grottenthaler 2004, Brion *et al*. 2008, Gale 2010). The Barker hypothesis suggests that the foetal development process is the origin of diseases in offspring (Barker 1995), and compromised pre-ovulation oocyte quality, especially related with epigenetic changes, may be crucial as well (Ge *et al*. 2014*a*). Epigenetics, including DNA methylation, histone modification and non-coding RNA, regulate gene expression by changing the conformation of chromosomes other than changing DNA sequences. However, epigenetic modifications may be affected by external and internal factors and the epigenetic changes may be inherited by daughter cells or the offspring (Flanagan *et al*. 2006, Bird 2007, Goldberg *et al*. 2007). Therefore, establishing proper genomic imprinting is a crucial event during oocyte maturation and early embryonic development in humans and other mammalian species. In the mouse female germline, DNA methylation is re-methylated during oocyte maturation after birth and it is completely established at the germinal vesicle (GV) stage. This process is mainly catalysed by DNA methyltransferase 3s (DNMT3s, reviewed by Tomizawa *et al*. (2012)). In mammalian oocytes, the reprogramming process takes place during oogenesis for histone modification and non-coding RNAs, too which is well discussed in previous reviews (Gu *et al*. 2010, Hales *et al*. 2011). Thus, if the internal and/or external factors are changed during oogenesis, the epigenetic reprogramming process may be disturbed. For instance, in female mice DNA methylation of imprinted genes in oocytes is altered by maternal diabetes and obesity (Ge *et al*. 2013*a*,*b*, 2014*b*). Furthermore, if one-carbon metabolism is abnormal, DNA methylation is changed in many tissues and it is correlated with diseases (Steegers-Theunissen *et al*. 2013).

Altered epigenetic modification may be an important factor for the complications seen in children of older mothers (Ge *et al*. 2014*a*,*b*). In this paper, we review the evidence for epigenetic alterations that have been detected in oocytes and that could be responsible for the age-related effects and the potential mechanisms.

## Ageing and epigenetic modifications in tissues

Damage to the reproductive and other systems increases with ageing in humans and in many other complex organisms, and there is a decrease in the adaptability and energy utilisation of the system. The accumulation of damage is caused by external and internal factors. Genetic factors only explain 20–30% of the variation in the human lifespan for twins and long-lived families, although these factors are crucial for survival to very old ages (Herskind *et al*. 1996, Mitchell *et al*. 2001, Poulsen *et al*. 2007). The other 70–80% of the variation may be caused by stochastic events, the environment and other non-genetic factors. Therefore, epigenetics, which is the link between the environment and genes and which regulates gene expression by mechanisms other than changes in the underlying DNA sequence (Goldberg *et al*. 2007), has been recognised as a possible contribution to the ageing phenotype (Wilson & Jones 1983, Fraga *et al*. 2005, McCauley & Dang 2015).

In mammals, epigenetic modifications are established during foetal development and most of them will be maintained throughout life by DNMT1. But the established epigenetic modifications in organs may be changed after birth if the external and/or internal environment is altered. The genome-wide result shows that global hypomethylation is associated with ageing (Liu *et al*. 2003, 2011). Genome-wide analysis shows that ageing is associated with a decrease in global genomic methylation, including CpG-poor promoters and tissue-specific genes (Heyn *et al*. 2012). Similar results have been obtained by other studies in blood and different tissues (Bjornsson *et al*. 2008, Moore *et al*. 2008, Bollati *et al*. 2009, Liu *et al*. 2009*a*, Kim *et al*. 2010, Gentilini *et al*. 2013, Wnuk *et al*. 2014). During the lifetime of monozygotic twins, the older monozygous twins exhibit remarkable differences not only in genomic distribution of 5-methylcytosine DNA but also in histone acetylation compared with the early years of life (Fraga *et al*. 2005). In the process of postnatal development and ageing of rhesus, dimethylation of histone 3 lysine 4 (H3K4me2) globally increases at promoters and enhancers (Han *et al*. 2012). Small non-coding RNA known as microRNA (miRNA) negatively modulates gene expression through binding to target mRNAs. The expression of miRNA is regulated by DNA methylation (Anwar & Lehmann 2014). Recently, the expression of miRNAs in ageing organs of mice and humans has also been observed (Pincus *et al*. 2011, Inukai *et al*. 2012, Ye *et al*. 2014). In older (>38 years) women, the miRNA profiling in the follicular fluid is clearly different compared with younger ones (<31 years) (Diez-Fraile *et al*. 2014). Although Anwar & Lehmann (2014) discussed that DNA methylation regulates the expression of miRNAs, the detailed mechanisms remained obscure. Besides DNA methylation, histone modifications and miRNAs, the chromatin structure and transposable elements in organisms are also altered with ageing in *Caenorhabditis elegans*, *Drosophila* and mouse model systems and the tissue culture-based replicative senescence model of cell ageing (Wood & Helfand 2013). Although the mechanism causing epigenetic changes in tissues after birth may be different from that in oocytes, the alterations in epigenetic modifications in different organs may indicate that epigenetic modifications in oocytes may be affected by ageing.

## Epigenetic changes in ageing oocytes

Herein, ‘ageing oocyte’ is defined as oocyte ageing that occurs in the ovaries of females who show a progressive decline in oocyte number and poor oocyte quality in ovaries during reproductive ageing (Tatone *et al*. 2008). The pregnancy rate of older women is lower compared with that of younger women, and the lower pregnancy rate (Menken *et al*. 1986) may mainly be caused by compromised oocyte quality (Wang *et al*. 2012). The child born to an older mother tends to exhibit the onset of some diseases in adulthood (Rocca *et al*. 1991, Kemkes-Grottenthaler 2004). These observations prompted us to ponder the mechanisms underlying this phenomenon. External and internal environmental alterations may cause changes in epigenetic modifications in oocytes. In animal models, the establishment of epigenetic modifications in oocytes is affected by maternal diets (Steegers-Theunissen *et al*. 2013), non-communicable diseases (Ge *et al*. 2013*b*) and other factors (Li *et al*. 2014). If the changes in epigenetic modifications occur in the germline, it would affect embryonic development and health of the offspring, and this effect may even extend to further generations (Ge *et al*. 2014*b*). Therefore, we discuss the association of oocyte quality, highlighting the epigenetic modifications, with maternal ageing.

### Ageing and oocyte quality

Many studies in the IVF–ET setting have demonstrated that ageing does not appear to affect the ability of oocytes to become fertilised, but compared with younger women, the implantation rates are lower and the spontaneous abortion rates are higher for older women (Romeu *et al*. 1987, Warburton *et al*. 1987, Lim & Tsakok 1997). Navot *et al*. (1991) reported that the age-related decline in female fertility is attributed to oocyte quality. For young mothers, about 20% of oocytes are aneuploid (Hassold *et al*. 2007), but the percentage increases to 50% or more in the oocytes of older mothers (Fragouli *et al*. 2011, Handyside *et al*. 2012). One reason may be that the level of cohesin, which is the key protein that regulates chromosome separation, falls below the level required to stabilise chiasmata and to hold sister centromeres tightly together in pre-ovulatory ageing oocytes (Lister *et al*. 2010, Jessberger 2012). Cohesion loss may be responsible for age-related meiotic segregation errors in mammalian oocytes (Petronczki *et al*. 2003, Revenkova *et al*. 2004, Tsutsumi *et al*. 2014). In humans, the expression of REC8 and SMC1B, the subunit of cohesin, in oocytes is decreased in 40-year-old women compared with 20-year-old women (Tsutsumi *et al*. 2014). There are other factors that may induce oocyte aneuploidy, which have been well reviewed recently (Jones & Lane 2013). Mitochondrial dysfunctions are observed in pre-ovulatory ageing oocytes (Dorland *et al*. 1998, Bentov *et al*. 2011, Eichenlaub-Ritter *et al*. 2011). As the amount of mitochondria decreases, the mutation of mitochondrial DNA may become increased and mitochondrial function is also affected in the oocytes of advanced-age female bovine, hamsters and mice (Eichenlaub-Ritter *et al*. 2011, Iwata *et al*. 2011, Simsek-Duran *et al*. 2013). Gene expression and chromatin structure (Wood & Helfand 2013) are also found to be affected by ageing. Hamatani *et al*. (2004) compared the difference in the expression profile at the transcript level of metaphase II (MII) oocytes of 5- to 6-week-old mice with that of 42- to 45-week-old mice. Among ∼11 000 genes whose transcripts are detected in oocytes, 5% showed obvious expression changes (Hamatani *et al*. 2004). Similar expression profiles in aged mouse oocytes have been reported in another study (Pan *et al*. 2008). An alteration in the expression profile of human MII oocytes is also associated with female ageing (Grondahl *et al*. 2010, Santonocito *et al*. 2013). In mouse oocytes, the mRNA and protein expression levels are altered with ageing, and dysfunctions of the ageing ovary may be a reason for the altered expression of mRNAs and proteins in pre-ovulatory ageing oocytes (Schwarzer *et al*. 2014, Tatone *et al*. 2014).

### Ageing oocytes and epigenetics

Previous discussions indicate that the epigenetic modifications of oocytes may be affected by advanced maternal age because the expression of DNMTs and histone acetyltransferases (*Myst1* (*Kat8*) and *Mrgx* (*Morf412*); Hamatani *et al*. 2004) is altered with ageing. Therefore, the changes in epigenetic modifications may partly explain why the child of an older woman is predisposed to the onset of hypertension, obesity and other diseases in adulthood (Aagesen *et al*. 1984, Malini & Ramachandra 2006).

#### Ageing oocytes and DNA methylation

If DNMT1, which maintains the DNA methylation patterns in oocytes and embryos (Mertineit *et al*. 1998), is deleted in mouse oocytes, the embryos show a loss of allele-specific expression and methylation at certain imprinted loci, and the foetuses of homozygous females die during the last third of gestation (Howell *et al*. 2001). In 5- to 6-week-old mouse oocytes, the gene expression profile is different from that in 42- to 45-week-old mouse oocytes. DNMT1, 3b and 3l are involved in the differential gene expression (Hamatani *et al*. 2004). Yue *et al*. (2012) found that the changes in genome-wide DNA methylation in oocytes and preimplantation embryos of 35- to 40-week-old mice were associated with decreased expression of DNMTs. The pregnancy rate of older Kunming mice (35–40 weeks old) is lower than that of younger mice, and the stillbirth and foetal malformation rate are higher in the older group compared with the younger group, which may be associated with abnormal DNA methylation in oocytes (Yue *et al*. 2012). In humans, TAP73 expression which is regulated by DNA methylation patterns is lower in the oocytes of women older than 38 years of age compared with the oocytes of women younger than 36 years of age (Guglielmino *et al*. 2011). There are still no direct proofs that the DNA methylation status in human oocytes is affected by ageing.

However, one study (Lopes *et al*. 2009) reports that the increase in resorption sites, morphological abnormalities and delayed development are related with the age of C57BL/6 mice (43–47 weeks old), but the monoallelic expression of the imprinted genes *H19* and *Snrpn* is normal in the blastocysts of aged female mice and the DNA methylation patterns of the differentially methylated regions (DMRs) of *Snrpn*, *Kcnq1ot1*, *U2af1-rs1* (*Zrsr1*), *Peg1*, *Igf2r* and *H19* are not altered. By Restriction Landmark Genome Scanning, the investigators also did not find significant differences in genome-wide DNA methylation in embryos and placentas from aged female mice (Lopes *et al*. 2009). This is contradictory to previous reports (Hamatani *et al*. 2004). The authors propose that the contradiction may be related to the materials selected and the limitation of the technique utilised in their study (Lopes *et al*. 2009). During zebrafish ageing, two CpG island shores are hypomethylated in oocytes, but they are *de novo* methylated in fertilised eggs (Shimoda *et al*. 2014). This suggests that the loss of methylation might be reset after fertilisation because there is a de-methylation and re-methylation process during early embryonic development.

Although reports about DNA methylation changes in oocytes from advanced-age females are contradictory, the popular viewpoint proposes that the DNA methylation in oocytes may be changed by pre-ovulatory ageing.

#### Ageing oocytes and histone modifications

Histone modifications, including methylation, acetylation, ubiquitination and other modifications, represent another crucial and well-investigated epigenetic modification. During meiosis, histone is deacetylated globally at the MI and MII stages by histone deacetylase (HDAC) activity in mammalian oocytes as revealed by immunostaining (Kim *et al*. 2003, Akiyama *et al*. 2004, Reddy & Villeneuve 2004). Akiyama *et al*. (2006) reported that if meiotic histone deacetylation was inhibited, aneuploidy occurred in fertilised mouse oocytes and this resulted in embryonic death in the uterus at an early stage of development. HDAC is downregulated at transcript level in ageing mouse (42- to 45-week-old) oocytes (Hamatani *et al*. 2004) although histone still remains acetylated in the oocytes of 10-month-old female mice (Akiyama *et al*. 2006). This suggests that histone modification in pre-ovulatory ageing oocytes may be affected (Table 1) and during development it may result in embryonic death. Similar results were obtained by Manosalva & Gonzalez (2009) and Suo *et al*. (2010). The expression of *Sirt2* which is related with the acetylation of histone H4K16 in the oocytes of aged mice is lower compared with that in younger mouse oocytes (Zhang *et al*. 2014*a*).

Another study found that the histone methylation in mouse GV oocytes was affected by advanced maternal age (Manosalva & Gonzalez 2010) (Table 1). Concomitantly, the GV and MII oocytes of older females lack H3K9me3, H3K36me2, H3K79me2 and H4K20me2 compared with the GV and MII oocytes of younger females. Meanwhile, the expression of the histone methylation-related factors (*Cbx1* and *Sirt1*) is changed in ageing GV oocytes. Histone 3 lysine 4 methylation in mouse GV oocytes is also changed by ageing (Shao *et al*. 2015).

In humans, the mRNA expression profile of MII oocytes is related with ageing. The differently expressed genes are involved in many biological processes, such as cell cycle, metabolism, apoptosis, protein modification and others (Grondahl *et al*. 2010). Recently, van den Berg *et al*. have shown that the histone acetylation staining of H4K5, H4K8, H4K12 and H4K16 was intensive in GV oocytes; however in MI and MII oocytes, chromatin was deacetylated in variable proportions. They also investigated the relationship between histone acetylation and maternal age. The results indicate that advanced maternal age negatively influences the deacetylation of H4K12 in human MII oocytes (van den Berg *et al*. 2011).

For women at aged 37–39 years, the gene for ubiquilin 1 (a ubiquitin-like protein) is downregulated in the oocytes, but three genes for the ubiquitin-specific peptidases *USP2*, *USP34* and *USP42* are upregulated (Grondahl *et al*. 2010). This suggests that the ubiquitination may be affected by age in human oocytes (Steuerwald *et al*. 2007). This indicates that histone ubiquitination might also be affected in oocytes by ageing, but there is still no solid evidence to confirm it.

#### Ageing oocytes and miRNA

miRNA is a kind of small non-coding RNA which functions in post-transcriptional regulation of gene expression upon recruitment into effector complexes (miRNA protein complexes or microRNPs; Truesdell *et al*. 2012). The post-transcriptional regulation may be particularly crucial for early mammalian development, from maturation of the germ line to initiation of gastrulation, because the genome is transcriptionally silent from the fully grown oocyte stage until zygotic genome activation (Abe *et al*. 2010). Small RNA is present in mouse oocytes, including miRNA (Tam *et al*. 2008), but Suh *et al*. (2010) suggest that miRNA function is globally suppressed during oocyte maturation and preimplantation development. However, loss of *Dicer*, which is crucial for the generation of endo-siRNA and miRNA, in mouse oocytes results in severe spindle and chromosomal segregation defects (Murchison *et al*. 2007), while loss of *Dgcr8*, which is essential only for miRNA processing, in the mouse has no effects on mRNA expression (Suh *et al*. 2010). Once *Ago2* is knocked out in mouse oocytes, the phenotype is similar to that observed in *Dicer*-knockout mouse oocytes (Kaneda *et al*. 2009). These results suggest that miRNAs function may be suppressed in mouse oocytes. However, miRNA mediates mRNA translation activation by FXR1 in *Xenopus laevis* oocytes (Truesdell *et al*. 2012). In mouse oocytes, miRNA-335-5p could affect oocyte maturation by regulating cytoskeleton dynamics (Cui *et al*. 2013). A similar result that miRNA-27a activation is not suppressed is observed in porcine oocytes (Chen *et al*. 2012). Many studies also demonstrated that miRNAs were essential for follicle development in different species (Abramov *et al*. 2013, Sohel *et al*. 2013, Yang *et al*. 2013, Zhang *et al*. 2014*b*). miRNAs expressed in oocytes could regulate bovine early embryogenesis (Tripurani *et al*. 2013). This contradiction has been well reviewed in previously published papers (Svoboda & Flemr 2010, Suh & Blelloch 2011).

It was shown that 79 miRNAs and 41 miRNAs existed in the microvesicles and exosomes isolated from equine follicular fluid respectively (Table 1), and three miRNAs are expressed significantly higher in exosomes isolated from follicular fluid of old mares compared with young ones (da Silveira *et al*. 2012). In humans, miRNAs are abundant in MII oocytes and cumulus cells and they may be essential for follicle development (Assou *et al*. 2013). Moreover, the miRNA expression profile in follicular fluid of women with polycystic ovary syndrome or premature ovarian failure is different from that in follicular fluid of unaffected women (Roth *et al*. 2014). In addition, miRNAs expression profiling of the follicular fluid of younger (<31 years) and older (>38 years) individuals was also investigated and the result showed that the expression of four miRNAs is different. These miRNAs are involved in carbohydrate digestion and absorption, p53 signalling and other biological processes that may be related with fertility. Therefore, this set of miRNAs and their respective targets should be evaluated in relationship with reproductive ageing (Diez-Fraile *et al*. 2014). However, the correlation between miRNA expression and oocyte quality during maternal ageing is still unknown.

#### Post-ovulatory ageing of oocytes and epigenetic modifications

After ovulation, when the arrested MII oocytes are not fertilised during the window of the optimal fertilisation time *in vivo* or *in vitro*, the unfertilised oocytes undergo a time-dependent decline concerning quality and this is called ‘post-ovulatory ageing of oocytes’ (Liang *et al*. 2012). Studies indicate that post-ovulatory ageing of mouse oocytes decreases the pregnancy rate, litter size and increases the percentage of male offspring compared with control females (Tarin *et al*. 1999, 2002, Kosubek *et al*. 2010, Liang *et al*. 2011). F1 offspring derived from post-ovulatory ageing oocytes are prone to the onset of growth retardation, delayed development of the righting reflex and emotionality (Tarin *et al*. 1999). Otherwise, post-ovulatory ageing of mouse oocytes decreases reproductive fitness and longevity of offspring (Tarin *et al*. 2002). This suggests that the epigenetic modification in post-ovulatory ageing of oocytes may be altered (Table 2). In our laboratory, we have analysed methylation patterns of imprinted genes in mouse oocytes during the post-ovulatory process. We examined methylation patterns of *Snrpn* and *Peg1* in *in vivo* and *in vitro* oocytes at 13, 21 and 29 h of human chorionic gonadotrophin (hCG) injection, respectively, and loss of methylation was observed at 29 h of hCG injection (Liang *et al*. 2008). Imamura *et al*. (2005) also reported *Peg1* lost methylation in oocytes during post-ovulatory ageing. However, only a small number of oocytes showed aberrant methylation in the DMR of *Peg3* in offspring derived from post-ovulatory ageing mouse oocytes (Liang *et al*. 2011). Although the DNA methylation of some imprinted genes is influenced by oocyte ageing, whether the whole-genome methylation patterns and histone modification are affected by oocyte post-ovulatory ageing is unclear. The histone modifications in post-ovulatory ageing of mouse oocytes are changed at 19 h of hCG injection compared with that at 14 h of hCG injection. When extending the time to 24 h of hCG injection, the fluorescence signals of acetylation of H3K14 also increased in oocytes (Huang *et al*. 2007). Another study reported that histone acetylation of H3K14 and H4K12 increased in mouse oocytes during post-ovulatory ageing (Liu *et al*. 2009*b*). This phenomenon is also observed in porcine oocytes during post-ovulatory ageing (Cui *et al*. 2011), although, the detailed underlying mechanism is still unknown.

#### Oocyte ageing and epigenetics: underlying mechanism(s)

##### Changes in enzymes related with epigenetic modifications

Epigenetic modifications are catalysed by numerous proteins, including DNMTs, ten-eleven-translocations (TETs), HDACs, ZFP57, Dicer, and KAP1/TRIM28. If their expression is affected in oocytes, the epigenetic modifications may be altered. DNMT1 and DNMT1o are crucial for maintaining proper methylation and DNMT3a, b and l are key *de novo* methylation enzymes. When the expression of DNMT1, which is necessary for maintaining DNA methylation, is disrupted in mouse oocytes, DNA methylation of imprinted genes is not maintained properly during early embryonic development (Hirasawa *et al*. 2008, Kurihara *et al*. 2008). The changed expression of DNMTs in oocytes from individuals of advanced maternal age (Hamatani *et al*. 2004, Grondahl *et al*. 2010) might be the direct reason for causing the DNA methylation alterations (Fig. 1). In MII oocytes of 35- to 40-week-old mice, the protein expression of DNMT1, DNMT3a, DNMT3b and DNMT3l is obviously lower than those in MII oocytes of 6- to 8-week-old mice (Yue *et al*. 2012). Anckaert *et al*. (2013) used 14-day *in vitro* follicle culture as a model to investigate pre-ovulatory intrafollicular oocyte ‘ageing’ and found that the mRNA levels of *Dnmt3a*, *Dnmt3l* and *Zfp57* were altered compared with 12-day *in vitro* follicle culture in the mouse.

The expression of enzymes related with histone modification is also affected by advanced maternal ageing in oocytes. For example, *Hdac2* is downregulated and the expression of histone acetyltransferases (*Myst1* and *Mrgx*) decreases in aged mouse oocytes (Hamatani *et al*. 2004). In old mice, the expression of the histone methylation-related factors *Cbx1* and *Sirt2* was changed in GV oocyte (Manosalva & Gonzalez 2010). The protein and kinase activities of CDC2A decreased in the GV and MII oocytes of old mice (Manosalva & Gonzalez 2009). Although specific inhibitors of HDACs could delay post-ovulatory oocyte ageing in mice and pigs (Huang *et al*. 2007, Jeseta *et al*. 2008, Lee *et al*. 2013), it is still unclear whether the change in histone acetylation is caused by the altered level or activities of HDACs for these post-ovulatory ageing oocytes from young mice.

Other enzymes related with epigenetic modifications, for example TETs (Yamaguchi *et al*. 2012), Trim28 (Messerschmidt *et al*. 2012) and Dicer (Murchison *et al*. 2007), may also be critical for oocyte developmental potential. However, there are few studies to investigate whether their expression in oocytes is affected by ageing or not. The proteins of the TET family are not only involved in DNA demethylation during early embryo development (Tahiliani *et al*. 2009, Gu *et al*. 2011, Ma *et al*. 2012), they are also crucial for female germ cells to complete meiosis (Yamaguchi *et al*. 2012). If the expression of *Tet3* is suppressed in oocytes, the paternal global demethylation process at the zygote stage is impaired (Gu *et al*. 2011). Although TETs are associated with ageing and diseases (van den Hove *et al*. 2012), a role for them in pre-ovulatory ageing oocyte and post-ovulatory ageing oocyte is still undetermined.

##### Changes in mitochondrial activity

Mitochondrial dysfunction may be another crucial factor inducing epigenetic changes in the oocytes of advanced females (Fig. 1). Oocyte maturation includes nuclear maturation and cytoplasmic maturation, and there are many biological events involved in these two processes, such as gene expression and histone and chromatin modifications (Eppig 1996). During oocyte maturation, energy (ATP) required is supplied by mitochondria (Torner *et al*. 2004). Histone and DNMTs use *S*-adenosyl-l-methionine (SAM) as a donor of methyl groups. SAM is biosynthesised using methionine and ATP (Igarashi & Katoh 2013). Wellen *et al*. (2009) demonstrated that ATP-citrate lyase was necessary for histone modification. Therefore, we conclude that if mitochondrial function is compromised by maternal age, the epigenetic modification may also be affected. In aged mouse oocytes, ATP and mitochondrial genomes are reduced to 38.4 and 44% respectively (Simsek-Duran *et al*. 2013). In bovine oocytes, the number of mitochondria and content of ATP are also affected by maternal age (Iwata *et al*. 2011). In post-ovulatory aged oocytes, reactive oxygen species (ROS) may be another factor inducing epigenetic changes. During *in vitro* ageing in porcine oocyte, H4K12 acetylation levels are related with ooplasmic ROS content (Cui *et al*. 2011). Glucose level is related with *de novo* purine and cAMP synthesis, which is associated with nuclear maturation of oocytes (Colton *et al*. 2003). The cumulus cells supply nutrition to oocytes in the final phase of oocyte maturation (Gilchrist *et al*. 2008). The function of cumulus cells is compromised by maternal age (Tatone & Amicarelli 2013). Thus, the energy transmitted from cumulus cells decreases, which may affect the establishment of epigenetic modifications in aged oocytes.

##### Nutrition effect

The changed nutrition supplies, especial one-carbon, may contribute to epigenetic changes in aged oocytes (Anckaert *et al*. 2010, Steegers-Theunissen *et al*. 2013; Fig. 1). For instance, the folate status declines with ageing, including decreased folate intake and altered folate availability (Jacob *et al*. 1998, Rampersaud *et al*. 2000). Therefore, the disruption of folate-mediated one-carbon metabolism by ageing may be another reason causing the abnormal DNA methylation in aged oocytes (Jacques *et al*. 2001).

##### Possible hormonal effects

A study demonstrated that oestrogen replacement therapy in menopause women reduced the total plasma homocystine concentration and increased genomic DNA methylation of mononuclear cells (Friso *et al*. 2007). In rodent brain, the methylation percent on the promoter of oestrogen receptor alpha is modulated by the expression of oestrogen (Schwarz *et al*. 2010). The DNA methylation is not only affected by hormones present in tissues, may be also in oocytes. In humans and mice, the methylation patterns of *Peg1* and *H19* are changed by hormones used for superovulation of growing oocytes (Sato *et al*. 2007). With ageing, the androgen level, which can be converted to oestrogen, is reduced for females (Blevins *et al*. 2013). The DNMT proteins and transcripts in the livers of 3-, 12-, and 24-month-old Ames dwarf mice are drastically reduced compared with WT siblings, and growth hormone appears to modulate the expression of DNMT1 and 3a (Armstrong *et al*. 2014). The amount of oestrogen which is important for follicular development decreases with ageing (Olsen & Kovacs 1996). Although there is no direct evidence showing that the epigenetic modifications are affected by hormones in pre- and post-ovulatory aged oocytes, it might contribute to the changes in epigenetic modifications in aged oocytes, which needs experimental validation (Fig. 1).

## Conclusion(s) and perspectives

The above discussions suggest that advanced maternal age and post-ovulatory oocyte ageing are deleterious to oocyte quality, including oocyte maturation, chromosome segregation, epigenetic modifications and mitochondrial function, and the health of the offspring from advanced-age mothers may also be affected by compromised oocyte quality (Takeo *et al*. 2013*a*). In this review, we mainly discussed the possible relationship between advanced maternal age and epigenetic modifications in oocytes and the potential underlying mechanisms. Two major problems are still unresolved: i) the detailed mechanisms underlying compromised oocyte quality including epigenetic changes caused by advanced maternal age and ii) the prevention of the adverse effects of oocyte ageing on epigenetic changes. As discussed previously, changes in enzymes including methyltransferases (DNMTs) and demethylases (TETs) may be the direct reasons for epigenetic alterations in aged oocytes, but whether/how ageing induces the changes in their expression needs further clarification. With the application of new technologies, it has been possible to test the transcriptome, global DNA methylation, histone modifications and proteome in limited number of cells or even in a single cell (Guo *et al*. 2014, Lovatt *et al*. 2014, Smallwood *et al*. 2014), which may help to address this issue. Another important study still to pursue is how to prevent the age-related deleterious effects on oocytes. If the disulphide-reducing agent dithiothreitol (DTT), an antioxidant, is supplemented to the culture medium, the negative effects of post-ovulatory ageing of mouse oocytes *in vitro* on fertilisation, cellular fragmentation at 24 h post-insemination and the potential of embryos for development until the blastocyst stage are prevented, at least in part (Tarin *et al*. 1998*b*). *N*-acetyl-cysteine (NAC) supplemented to medium can also decrease ROS levels in post-ovulatory aged oocytes, but only resveratrol increased the fertilisation rate (Takeo *et al*. 2013*b*). Whether the epigenetic changes in these oocytes are prevented is unknown. But another study demonstrated that if adult female mice were subjected to caloric restriction, they did not exhibit age-related increases in oocyte aneuploidy, meiotic spindle abnormalities or mitochondrial dysfunction, all of which occurred in the oocytes of age-matched controls (Selesniemi *et al*. 2011). This indicates that the epigenetic modification in aged oocytes may also be affected by calorie restricted diet because energy is crucial for epigenetic modifications. In medium supplemented with pyruvate, post-ovulatory oocyte ageing is prevented and changes in histone acetylation are corrected in the mouse oocyte (Liu *et al*. 2009*b*). Some studies suggest that both DNA methylation and histone modification are associated with caloric restriction (Li *et al*. 2011, Chouliaras *et al*. 2012, Chen *et al*. 2013). One-carbon supplies methyl for methylation, so changing nutrients in diet might prevent abnormal methylation in oocytes. If the levels of methyl donor are lower in the medium during mouse follicle culture, the establishment of oocyte imprinting is affected (Anckaert *et al*. 2010). Steegers-Theunissen *et al*. (2013) reviewed the effects of one-carbon on reproduction and long-term health of offspring. These studies suggest that age-related effects, including epigenetic changes, on oocytes might be prevented by diets, medicine or other methods. However, until now we still cannot effectively prevent the age-related deleterious effects on oocytes.

## Figures and Tables

**Table 1:** Effects of advanced maternal age on epigenetics in oocytes.

**Species**	**Epigenetic changes**	**References**
Mouse	Genome-wide DNA methylation is lower in 35- to 40-week-old mouse oocytes	Yue *et al*. (2012)
Mouse	DNMTs (DNMT1, 3a, 3b and 3l) expression is decreased in aged mouse oocytes	Hamatani *et al*. (2004) and Pan *et al*. (2008)
Mouse	Histone deacetylase is downregulated and histone remains acetylated in older mouse oocytes. Histone acetylation of H4K12 is affected in aged GV and MII oocytes	Hamatani *et al*. (2004), Akiyama *et al*. (2006), Manosalva & Gonzalez (2009) and Suo *et al*. (2010)
Mouse	*Sirt2* expression is lower in the oocytes of old mice compared with young mice	Zhang *et al*. (2014*a*,*b*)
Mouse	H3K9me3, H3K36me2, H3K79me2 and H4K20me2 are altered in aged oocytes	Manosalva & Gonzalez (2010)
Mouse	The expression of *Cbx1* and *Sirt1* is changed in the oocytes of older mice	
Mouse	Histone 3 lysine 4 methylation is changed in aged GV oocytes	Shao *et al*. (2014)
Bovine	Non-imprinted genes (*SLC2A1*, *PRDX1*, *ZAR1* and *BTS*) are hypomethylated in the oocytes of adult cows compared with prepubertal calves	Diederich *et al*. (2012)
Zebrafish	Two CpG island shores hypomethylated in oocytes with ageing	Shimoda *et al*. (2014)
Human	The deacetylation of H4K12 in human MII oocytes is affected at an age-dependent manner	van den Berg *et al*. (2011)
Human	The expression of ubiquilin I, *USP2*, *USP34* and *USP42* is affected in the oocytes of women aged 37–39 years	Grondahl *et al*. (2010)
Human	MicroRNAs expression profiling of the follicular fluid of younger females is different from that of the follicular fluid of older females	Diez-Fraile *et al*. (2014)
Equine	Three miRNAs are expressed in significantly higher amounts in exosomes isolated from follicular fluid of old compared to young mares	da Silveira *et al*. (2012)

**Table 2:** Effects of post-ovulatory ageing of oocytes on epigenetic modifications.

**Species**	**Epigenetic changes**	**References**
Mouse	DNA methylation patterns of *Snrpn* and *Peg1* in oocytes is altered at 29 h after hCG injection	Liang *et al*. (2008)
Mouse	*Peg1* loss of methylation in oocytes during post-ovulatory ageing	Imamura *et al*. (2005)
Mouse	Acetylation of H4K8, H4K12 and H3K14 is altered in ageing oocytes	Huang *et al*. (2007) and Liu *et al*. (2009*b*)
Porcine	Histone of H4K12 is changed in post-ovulatory ageing oocytes	Cui *et al*. (2011)
